# Supplementary material for: Chemoproteomic Elucidation of β‑Lactam Drug Targets in Mycobacterium abscessus
Source: ACS Infect Dis. 2026 Apr 17;12(5):1669–80. doi: 10.1021/acsinfecdis.6c00011 (PMC13162264; doi:10.1021/acsinfecdis.6c00011)
Supplement: Supplementary file 3 [file id6c00011_si_003.pdf]

Supporting Information for:

Chemoproteomic elucidation of β-lactam drug targets in *Mycobacterium abscessus*

Kaylyn L. Devlin[1], Emily Hutchinson[1], Damon T. Leach[2], Leo J. Gorham[3], William C. Nelson[2], Gyanu Lamichhane[4], Vivian S. Lin[2,5], and Kimberly E. Beatty[1*]

1. Department of Chemical Physiology and Biochemistry, Oregon Health & Science University, Portland, Oregon 97239
2. Biological Sciences Division, Pacific Northwest National Laboratory, Richland, Washington 99354
3. Nuclear Sciences Division, Pacific Northwest National Laboratory, Richland, Washington 99354
4. Division of Infectious Diseases, School of Medicine, Johns Hopkins University, Baltimore, Maryland 21231
5. Institute of Biological Chemistry, Washington State University, Pullman, Washington 99164

* Corresponding author: beattyk@ohsu.edu

Mycobacterial strains

Mycobacterium abscessus (*Mab*) strains used in the present study include lab strain ATCC 19977 (subsp. *abscessus*) and a clinical isolate M9510[1, 2] (subsp. *massiliense*). Strains were obtained from the G. Lamichhane laboratory (Johns Hopkins University) and handled as BSL-2 pathogens.

Mycobacterial Culture and Measurement of Colony Forming Units

Mycobacteria were thawed from frozen stocks stored at -80 °C in 30% glycerol. Routine *Mab* culture was done in LGM1 medium (**Table S1**) at 37 °C with 100 rpm in aerated polycarbonate shake flasks with a 0.2 µm filter cap (Weaton #WPFPC0500S).

Culture conditions to induce a non-replicating state via nutrient starvation were optimized from previous reports[3-5]. Carbon starvation (CS) and total nutrient starvation (NS) cultures (n = 3-5, 20 mL) were prepared from exponential phase *Mab* 19977 (grown in LGM1) washed twice in PBS and diluted (OD_{600} of 0.2) in either Middlebrook 7H9 broth, 0.05% tyloxapol (CS) or PBS, 0.05% tyloxapol (NS). Samples were cultured standing at 37 °C in 150 mL plug-seal bottles for eight days. Comparative standard (high nutrient) cultures (n = 3) were prepared at a starting OD_{600} of 0.05 in aerated shake flasks and cultured in LGM1 at 37 °C with 100 rpm. Starvation culture conditions were assessed by measuring OD_{600} and colony forming units (CFUs) (**Figure S1**). CFUs were measured by plating 10-fold serial dilutions of cultures on 7H11-S agar plates (**Table S1**) in triplicate. Colonies were counted four days after plating.

Carbon starved and replicating cultures of *Mab* 19977 and M9510 were prepared for proteomic analysis. *Mab* were grown to an OD_{600} of ~0.7 in LGM1, washed twice in PBS, and diluted (OD_{600} of 0.2) in carbon starvation medium (7H9-Tx, **Table S1**). Cultures (CS, n = 6, 300 mL) were grown standing at 37 °C in 1 L plug-sealed bottles (Corning #430195) for 72 hrs. Matched replicating cultures were simultaneously

prepared from the same washed cell stock after dilution (OD_{600} of 0.2) in LGM1-Tx medium (**Table S1**). Replicating cultures (Rep, n = 6, 200 mL) were grown shaking (100 rpm, 37 °C) for several hours in aerated 500 mL shake flasks until an OD_{600} of ~0.4 was reached. Cells were harvested through centrifugation (5 min, 4000 xg, 4 °C), washed twice with PBS, and stored at -30 °C in PBS until lysis.

Preparation of *Mab* Lysates
Cells were lysed as previously described[6]. Briefly, whole cell lysates were obtained by mechanical disruption in phosphate-buffered saline (PBS) and then extracted in 1% n-dodecyl-D-β-maltoside (Chem-Impex #21950, CAS 69227-93-6) in PBS (PBS-DM). Lysates were filtered twice through 0.2 µm PVDF membrane filters (13 mm, Pall) to sterilize. A bicinchoninic acid (BCA) assay (Pierce) was used to quantify total protein concentration of all lysates.

Imaging of β-lactam Target Labeling in Live *Mab* Cells
Mab 19977 cells were pelleted (4000 xg, 3 min, 4 °C), washed in PBS, and treated with 4 µg/mL avibactam (Advanced ChemBlocks, CAS 1192500-31-4) in PBS (30 min, 37 °C, standing). Cells were washed (PBS) and labeled with 10 µM Mero-sCy5, 10 µM Bocillin FL, and 5 µg/mL DAPI (30 min, 37 °C, standing). Labeled cells were washed (PBS) before fixation in 4% paraformaldehyde (1 hr, RT). Cells (2 µL) were spotted on cover glass (Zeiss, #1.5), air dried, and mounted on a glass slide with ProLong Gold Antifade mountant. Samples were stored at 4 °C until imaged.

Samples were imaged on a Zeiss Elyra 7 microscope using lattice structured illumination (SIM) and SIM^2 processing. The following excitation (ex) lasers and emission (em) filters were used for the specified fluorophore: DAPI- ex 405 nm, em 420-480 nm; Bocillin FL- ex 488 nm, em 495-550 nm; Cy5- ex 642 nm, em LP 655. Micrographs were taken using a 63x oil-immersion lens (1.4 NA, Zeiss). Micrograph brightness/contrast settings were adjusted in Fiji.

In-Gel Analysis of Mero-sCy5 and Bocillin FL Binding
Normalized total protein lysates (30 µg) were labeled with 10 µM Meropenem-sulfoCy5[6] (Mero-sCy5) or 10 µM Bocillin FL (Invitrogen) for 60 min at RT, protected from light. Labeling reactions were quenched by the addition of 1x TCEP loading dye (5X stock: 10% SDS, 289 mM Tris, 50 mM TCEP, 0.025% [w/v] Ponceau S Red, and 63% glycerol). Samples were heated to 75 °C for 10 min and resolved (12 µg/lane) via SDS-PAGE (Criterion Bis-Tris gels, XT-MES running buffer, Bio-Rad). Gels were de-stained of free probe overnight (30% methanol, 10% acetic acid in water) and scanned using Cy5 (ex 635, em 670BP30) and Cy2 (ex 488, em 525BP20) laser/emission filters on an Amersham Typhoon imager (Cytiva). Adjustment of image brightness and contrast and band intensity quantification were performed with Fiji[7]. Total protein staining was done using Coomassie R-250 (Thermo Scientific, CAS 6104-58-1) (**Figure S3**).

Labeling and Enrichment of Lysates for ABPP
Affinity enrichment of meropenem-biotin (Mero-biotin) labeled *Mab* lysates was done as previously described[6]. Lysates (600 µg total protein) were incubated with 30 µM Mero-

biotin or vehicle (no probe control [NPC], n=4) (60 min, RT). Excess Mero-biotin was removed via 3 kDa molecular weight cutoff (MWCO) filtration (Amicon). Biotinylated proteins were affinity purified on streptavidin-agarose resin (Thermo Fisher Scientific, 20353). Samples were bound to prewashed resin in 1% SDS in PBS at a protein-to-resin ratio of 4:1 (µg:µL) (60 min, RT, end-over-end rotation). Resin-bound protein was washed within fritted chromatography columns (Bio-Rad, 7326008) with 13 column volumes as follows: 1x 1% SDS in PBS, 3x 0.5% SDS in PBS, 1x 6 M urea in 25 mM ammonium bicarbonate, 3x ultrapure water, 4x PBS, 4x 25 mM ammonium bicarbonate (pH 8). Resin-bound protein was digested with sequencing-grade trypsin (Promega, V5111) at a trypsin:protein ratio of 1:4000 (µg:µg) (overnight, RT, end-over-end rotation). Digested peptides were separated from resin (2000 xg, 5 min, RT) and residual peptides were collected off resin with additional 25 mM ammonium bicarbonate (30 min, RT, end-over-end rotation). All samples were stored at -20 °C before transfer to Pacific Northwest National Laboratory (PNNL) for LC-MS/MS analysis.

Proteomics samples were analyzed by western blot to assess sample quality (**Figure S9**). After labeling, aliquots of Mero-biotin and NPC samples were denatured in 1X TCEP SDS-PAGE loading dye by heating (10 min, 75 °C). Normalized protein (5 µg) was resolved via SDS-PAGE on a 4-12% Bis-Tris gel (Bio-Rad, 1X XT MOPS running buffer). Proteins were either transferred to a membrane for western blot analysis or stained with Coomassie G-250 (Fisher, CAS 6104-59-2). Stained gels were imaged on a flat-bed scanner (Epson).

Resolved protein for western blot analysis were transferred to a PVDF membrane (100 V, 60 min). Blots were blocked with 5% bovine serum albumin (BSA) in 1X TBST (1 M TBS, 0.1% Tween 20) (18 h, 4 °C). Blots were washed 3x in 1X TBST and incubated with streptavidin-HRP (Thermo Scientific S911, 1:3000) in 5% BSA in 1X TBST (30 min, RT). Blots were washed 3x in 1X TBST, developed with SuperSignal West Pico PLUS (Thermo Scientific, 34580), and imaged on a MyECL Imager (Thermo Scientific) (**Figure S9**). The brightness and contrast of the acquired images were adjusted in Fiji.

Mass Spectrometry Sample Preparation

Peptides were evaporated to dryness in a ThermoFisher SpeedVac vacuum concentrator. Dried peptides were reconstituted in 40 µL of 25 mM ammonium bicarbonate and heated to 37 °C for 5 min at 1000 rpm on a thermoshaker. Samples were briefly centrifuged to pellet insoluble debris and the clarified supernatant transferred to polycarbonate ultracentrifuge tubes. Samples were centrifuged at 53,000 rpm in a Beckman ultracentrifuge with TLA 120.1 rotor for 20 min at 4 °C. After centrifugation, 25 µL of supernatant was carefully transferred to LC-MS vials and stored at -20 °C until ready for LC-MS/MS analysis.

Liquid Chromatography Tandem Mass Spectrometry (LC-MS/MS)

ABPP samples were analyzed using a Waters nanoAcquity ultra performance liquid chromatography (UPLC) system and Thermo Scientific Q Exactive Plus Orbitrap mass spectrometer. Samples were loaded into a precolumn (150 µm i.d., 4 cm length, packed in-lab with Jupiter C18 packing material, 300 Å pore size, 5 µm particle size;

Phenomenex) using mobile phase A (0.1% formic acid in water). The separation was carried out in a NanoLC column (75 µm i.d., 30-cm column, CoAnn Technologies), packed in-lab with BEH C18 packing material (130-Å pore size, 1.7 µm particle size, Waters Corporation). Chromatography was performed with a flow rate of 200 nL/min using a 60 min gradient of 1-75% mobile phase B (acetonitrile + 0.1% formic acid). The column was washed with 95-50% mobile phase B for 20 min and equilibrated with 1% mobile phase B for 30 min before each sample injection to prevent carryover. The mass spectrometer source was set at 2.2 kV, and the ion transfer capillary was heated to 300 °C. Data-dependent acquisition (DDA) mode was employed to automatically trigger the precursor scan and the MS/MS scans. The MS1 spectra were collected at a 300-1800 m/z scan range, a resolution of 70,000, an automatic gain control (AGC) target of 3 x 10^6, and a maximum ion injection time of 20 ms. For MS2, the top 12 most intense precursors were isolated with a window of 1.5 m/z and fragmented by higher-energy collisional dissociation (HCD) with a normalized collision energy at 30%. The Orbitrap was used to collect MS/MS spectra at a resolution of 17,500, a maximum AGC target of 10^5, and maximum ion injection time of 50 ms. Each parent ion was fragmented once before being dynamically excluded for 30 s.

Analysis of Mass Spectrometry Data

MS/MS Automated Selected Ion Chromatogram generator (MASIC) was used to generate selected ion chromatograms (SICs) for all of the parent ions chosen for fragmentation in the LC-MS/MS data[8]. MSGF+ software[9] was then used to perform peptide searches against the *M. abscessus* ATCC 19977 protein database (UniProt taxon ID 561007, downloaded on 2024-01-15) with the following parameters: parent ion tolerance of 20 ppm; methionine oxidation (+15.9949 Da) as a dynamic modification. M9510 peptides were searched against the protein FASTA (generated using Prokka software v. 1.14.6) for *Mycobacteroides abscessus* M9515 (subsp. *massiliense*)[2]. Mage software (v. 1.5.8987, https://github.com/PNNL-Comp-Mass-Spec/Mage) was used to extract the MSGF+ first hits results and export data for further processing. Story-Roller et al.[2] described the genomes of these strains but several unresolved gaps prevented complete assembly of the M9510 genome. Alignment of M9510 contigs against the M9515 chromosome revealed near-perfect sequence identity across the genome. Because of this high concordance and the absence of substantive genomic differences, the complete M9515 genome sequence was used as the reference for proteomic analyses of M9510.

ABPP analyses were conducted using chemoprotR, a custom R package for ABPP data processing based on pmartR[10] (https://github.com/pmartR/chemoprotR). Data were filtered such that only peptides with an MS-GF+ spectral probability of less than 1.56 x 10^{-8} were retained, based on calculations for a 1% false discovery rate (FDR) from *M. tuberculosis* global proteomics data using a target-decoy approach[6, 11]. Once filtered, peptide redundancies were removed by summing reporter ion masses, and reporter ion values were log2 transformed. Data were then normalized using mean centering with a group-specific backtransformation[12, 13]. Peptide-level data were rolled up to a protein-level using the method, “rollup”[14]. We observed that the method used for peptide to protein summarization may impact results, and additional consideration of the method

used is recommended[15]. Once rolled up to the protein level, reverse hits and contaminants were removed from the dataset.

Statistical differences in protein intensity between ABPP groups (ABP vs. NPC) was assessed via analysis of variance (ANOVA) and independence of missingness (IMD, G-test)[16]. In cases when a protein was observed in at least two samples in each group, it was considered significantly different if the mean log2 intensity between groups had a $p \leq 0.05$ by ANOVA. In cases when a protein was observed in less than two samples in one group, the number of observed values between groups was considered statistically different if $P \leq 0.05$ by IMD. To identify a protein as specifically labeled by meropenem-biotin, the following criteria were applied: (1) present in at least 3 ABP replicates, (2) a difference between ABP sample and NPC mean intensity with $p < 0.05$ (IMD-ANOVA), (3) a 3-fold higher intensity in ABP samples relative to NPC samples. Proteins known to be biotinylated or biotin-binding were manually removed from the lists (**Table S3**). Comprehensive lists of identified targets are included in **Table S2** and are deposited in the Mass Spectrometry Interactive Virtual Environment (MassIVE) repository accession: MSV000100028 (https://massive.ucsd.edu/ProteoSAFe/dataset.jsp?task=bf3e1f148c94484bb2118f350eeaaac2).

Ortholog Mapping

Proteome datasets for *Mtb* H37RV and *Mab* 19977 were downloaded from the UniProt[17] proteome database on 2024-06-27. The *Mab* M9515 proteome was obtained from the Lamichhane laboratory (personal communication). The proteomes were analyzed using OrthoFinder[18] (v 3.1.0), OrthoMCL[19] (v 2.0.9), the MMSEQS2[20] (v 13.45111) easy-cluster function, and reciprocal best hits analysis based on BLAST+[21] (v 2.12.0) searches and a modified version of Multiparanoid[22]. Results from all searches were parsed and clusters of proteins with similar membership profiles were generated using bespoke code (code available upon request).

Expression and Native Purification of *Mab* Enzymes

Plasmids for the expression of *Mab* enzymes (**Table S5**, pET-28a+-TEV vector) were designed to exclude N-terminal transmembrane and signal sequences and were purchased from Genscript.

All enzymes were expressed in 100 mL cultures (2XYT media supplemented with kanamycin) of BL21-Star-(DE3) cells (Invitrogen) and induced with 250 μM IPTG at 20 °C for 18 hrs. Cells were lysed by sonication in 50 mM Tris-Cl pH 8.0 at 4 °C, 150 mM NaCl, 20 mM imidazole, 1 mg/mL lysozyme, 1% sarkosyl (w/v) and pelleted (10,000 xg, 20 min, 4 °C). Clarified lysate was bound to Ni-NTA resin (ThermoFisher, 2 mL, 4 °C, 60 min) and purified under native conditions according to the Qiagen QIAexpressionist Handbook (5th edition). Briefly, resin was washed with 30 column volumes of 50 mM NaH_2PO_4 pH 8, 500 mM NaCl, 20 mM imidazole and eluted in 9 column volumes of 50 mM NaH_2PO_4 pH 8, 500 mM NaCl, 250 mM imidazole. Recombinant protein in elution fractions was concentrated and buffer exchanged into 50 mM Tris-Cl pH 8.0 at 4 °C,

150 mM NaCl using Amicon Ultra 10K MWCO centrifugal filters (Millipore). Enzyme purity was assessed by SDS-PAGE (Figure S6) and enzyme concentration was measured by BCA assay (Pierce). Enzymes were stored at 4 °C and used without further purification.

Measurement of β-lactamase Activity

β-lactamase activity was assessed through measurement of nitrocefin (EMD Millipore, CAS 41906-86-9) hydrolysis. Purified enzymes (8 µM) or buffer-only control were incubated with 200 µM nitrocefin in 50 mM Tris-Cl (pH 8.0 at 4 °C), 150 mM NaCl for 2 hr at 37 °C in a 96-well microplate (Corning 3695). Immediately following substrate addition, nitrocefin hydrolysis was monitored at 486 nm on a microplate reader (Tecan, 45 s intervals). Endpoint absorbance values were background corrected by subtraction of the respective sample's absorbance at assay initiation. Corrected 2 hr absorbance values were converted to amount of hydrolyzed nitrocefin using Beer's law (ε_{486} = 20,500 $M^{-1}cm^{-1}$). All calculations were done using technical triplicates from two separate experiments. The statistical difference between nitrocefin hydrolyzed by each enzyme compared to buffer alone was assessed by multiple unpaired t-test using GraphPad Prism software (10.2.0) and error was calculated as standard deviation.

Kinetics Analysis

Purified enzyme (Ldt4, 5 µM; PbpB, 20 µM; MAB_3998, 20 µM) or buffer alone was combined with nitrocefin (Ldt4, 10-200 µM; PbpB, 50-1000 µM; MAB_3998, 25-500 µM) in a 96-well microplate in 50 mM Tris-Cl pH 8.0 at 4 °C, 150 mM NaCl. Nitrocefin hydrolysis was monitored at 486 nm on a microplate reader (30 sec intervals, 1-2 hr duration, 37 °C). For Ldt4 and PbpB, initial rates were calculated from 240-570 sec of the reaction. For MAB_3998, the initial rates were calculated from 120-1200 sec of the reaction. The velocity against substrate concentration was fit to a nonlinear regression assuming Michaelis-Menton steady-state kinetics in GraphPad Prism software. Calculations were done using technical triplicates from two (MAB_3998) or three (Ldt4, PbpB) separate experiments. Error is reported as standard deviation.

β-lactam Competition of ABP Labeling

Recombinant enzymes

Purified enzymes (3 µg) were incubated with 500 µM imipenem monohydrate (IPM, MedChemExpress, CAS 74431-23-5), cefoxitin sodium salt (FOX, Sigma-Aldrich, CAS 33564-30-6), avibactam free acid (AVI, Advanced Chemblocks, CAS 1192500-31-4), or buffer only in 10 mM HEPES (pH 7.5) for 30 min at 37 °C. Samples were then incubated with 10 µM Mero-sCy5 or Bocillin FL for 60 min at 37 °C. Samples were denatured with 1X TCEP SDS-PAGE loading dye by heating (10 min, 75 °C). Protein (1 µg/lane) was resolved via SDS-PAGE on 10% Bis-Tris gels (Bio-Rad, 1X XT MES running buffer). Triplicate samples of each enzyme were prepared independently and run within the same gel to standardize background. Gels were destained of free probe overnight (30% methanol, 10% acetic acid in water) and scanned using Cy5 and Cy2 laser/emission filters on an Amersham Typhoon imager (Cytiva). Total protein staining was done using Coomassie R-250 (Thermo Scientific, CAS 6104-58-1).

Adjustment of image brightness and contrast and band intensity quantification was performed with Fiji. Statistical comparison of mean band intensities between no treatment and drug-treated enzymes was done using one-way ANOVA with Tukey multiple comparison test in GraphPad Prism software.

Lysates

Mab 19977 and M9510 whole-cell lysates were incubated with 1 mM IPM, FOX, AVI, meropenem trihydrate (MER, MedChemExpress, CAS 119478-56-7), penicillin G sodium salt (PEN, Thermo Scientific, CAS 69-57-8), aztreonam (AZT, aablocks, CAS 78110-38-0), or buffer only in 10 mM HEPES (pH 7.5) for 30 min at 37 °C. Samples were then incubated with 5 μM Mero-sCy5 or buffer only for 60 min at 37 °C. Samples were denatured with 1X TCEP SDS-PAGE loading dye by heating (10 min, 75 °C). Protein (5 μg/lane) was resolved by SDS-PAGE on 4-12% Bis-Tris gels (Bio-Rad). Gels were destained of free probe overnight and scanned using Cy5 laser/emission filters on an Amersham Typhoon imager.

β-lactam IC_{50} Measurement

We determined the inhibitory concentration 50 (IC_{50}) of imipenem and cefoxitin for PbpB, Ldt4, DacB2, and MAB_4800. We measured the IC_{50} by drug-treatment followed by Bocillin FL labeling using the method described by Carlson[23]. Purified enzyme (0.5 μM) was treated with a range of inhibitor concentrations (.01 – 5000 μM) or buffer only (NT) in 50 mM Tris-Cl (pH 8.0 at 4 °C), 150 mM NaCl for 30 min at 37 °C. Samples were then incubated with 10 μM Bocillin FL for 30 min at 37 °C. Reactions were stopped by addition of 1X TCEP SDS-PAGE loading dye and heating (5 min, 75 °C). Technical duplicates were loaded and resolved on the same gel to standardize background. Gels were destained of free probe overnight (30% methanol, 10% acetic acid in water) and scanned using the Cy2 laser/emission filter on an Amersham Typhoon imager. The experiment was repeated twice for each enzyme.

Mean band intensities were measured in ImageJ by drawing a box tightly around the band. The inhibitor concentration versus mean band intensity was plotted and fit to a nonlinear regression ("Dose-response Inhibition"; variable slope 4 parameters) in GraphPad Prism software. Final values were calculated by averaging the IC_{50} across the two biological replicates, using error propagation to calculate the standard error. An example of this analysis is shown in **Figure S8**.

We analyzed MAB_0330 and MAB_2833 using this method but were unable to fit the data in GraphPad Prism to calculate an accurate IC_{50}. For MAB_0330, the error was unacceptably large (e.g., 170 μM ± 180 μM for IPM and 270 μM ± 240 μM for FOX). For MAB_2833, there was little change in band intensity upon drug treatment, leading to poor data fitting.

Supplementary Figures

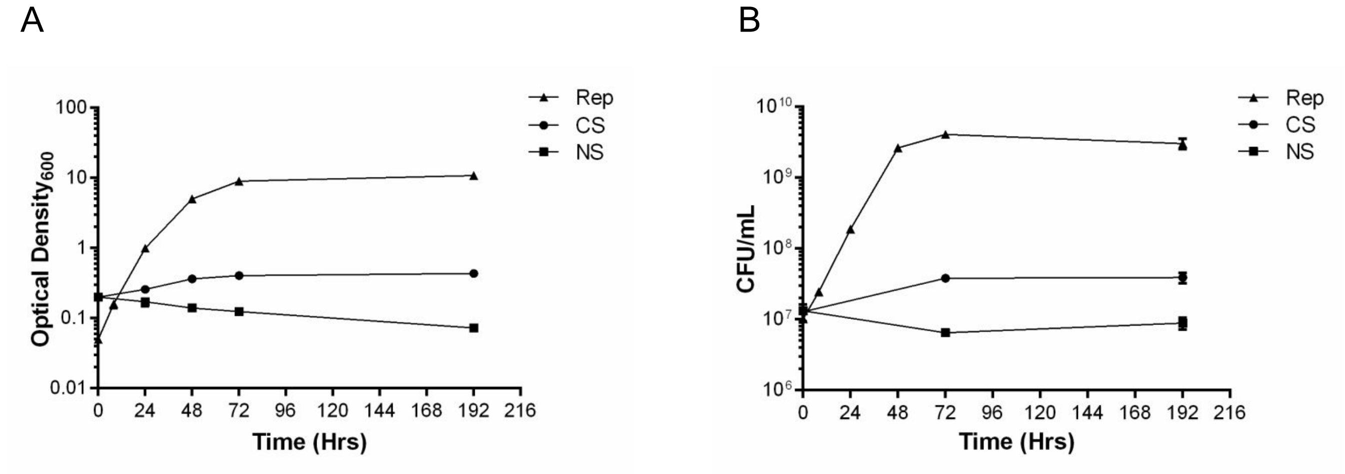

Figure S1. ***Mab*** **19977 growth under starvation conditions**. *Mab* 19977 were cultured in high-nutrient (Rep), carbon starved (CS), or total nutrient starved (NS) conditions for eight days. Optical density at 600 nm (OD_{600}) (**A**) and colony-forming units per mL (CFU/mL) (**B**) were measured at specified time points.

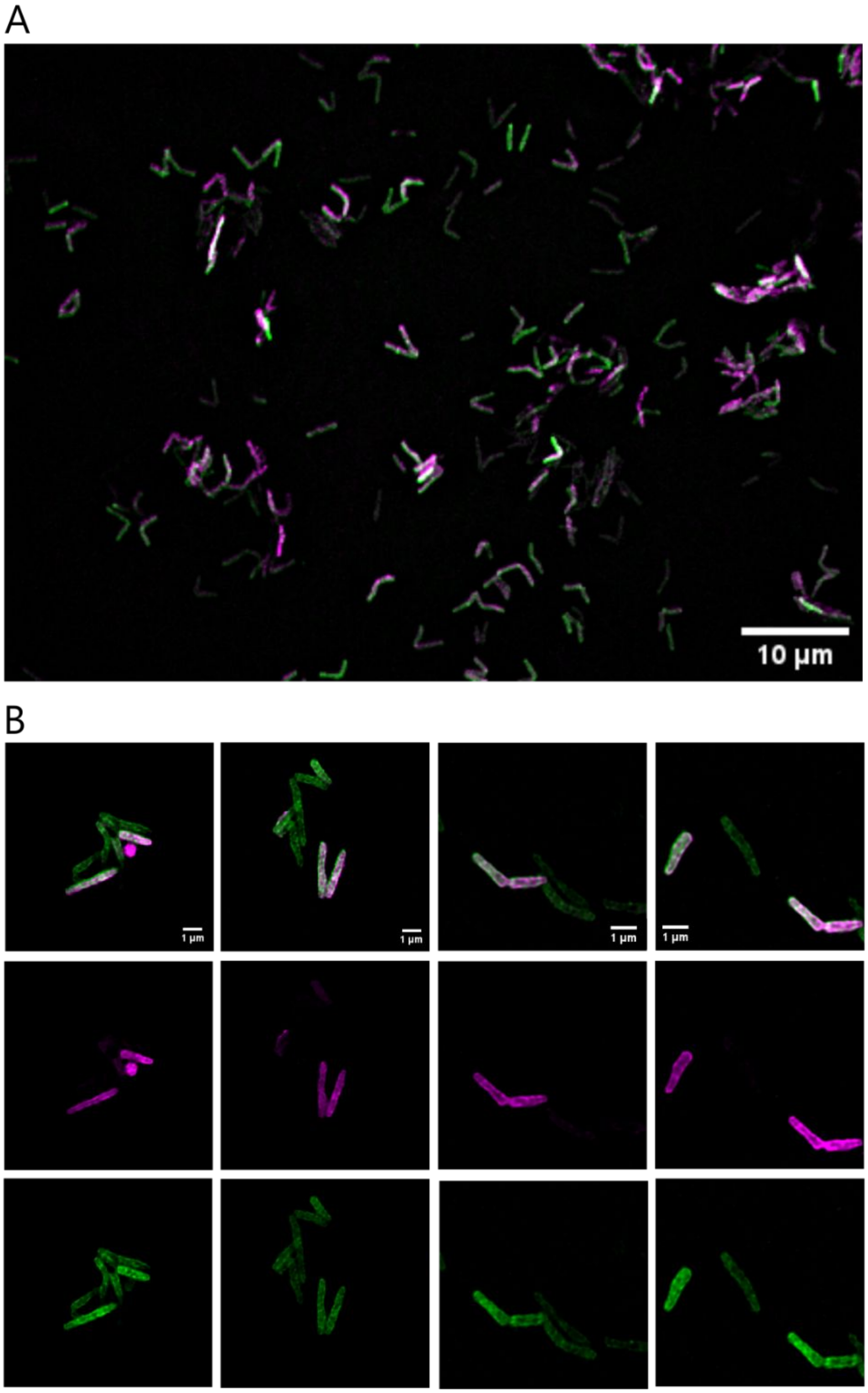

Figure S2. Super resolution imaging of *Mab* cells labeled with β-lactam probes. Live *Mab* 19977 cells were labeled with Mero-sCy5 (10 μM) and Bocillin FL (BFL, 10 μM), fixed, and mounted. Samples were imaged using structured illumination microscopy using Cy5 (Mero-sCy5) and Cy2 (BFL) laser and filter settings. Micrographs were false-colored (Cy5, magenta; Cy2, green) and channels were merged (overlap appears white). Cropped micrographs of representative cells from a field of view (**A**) are shown in **B**.

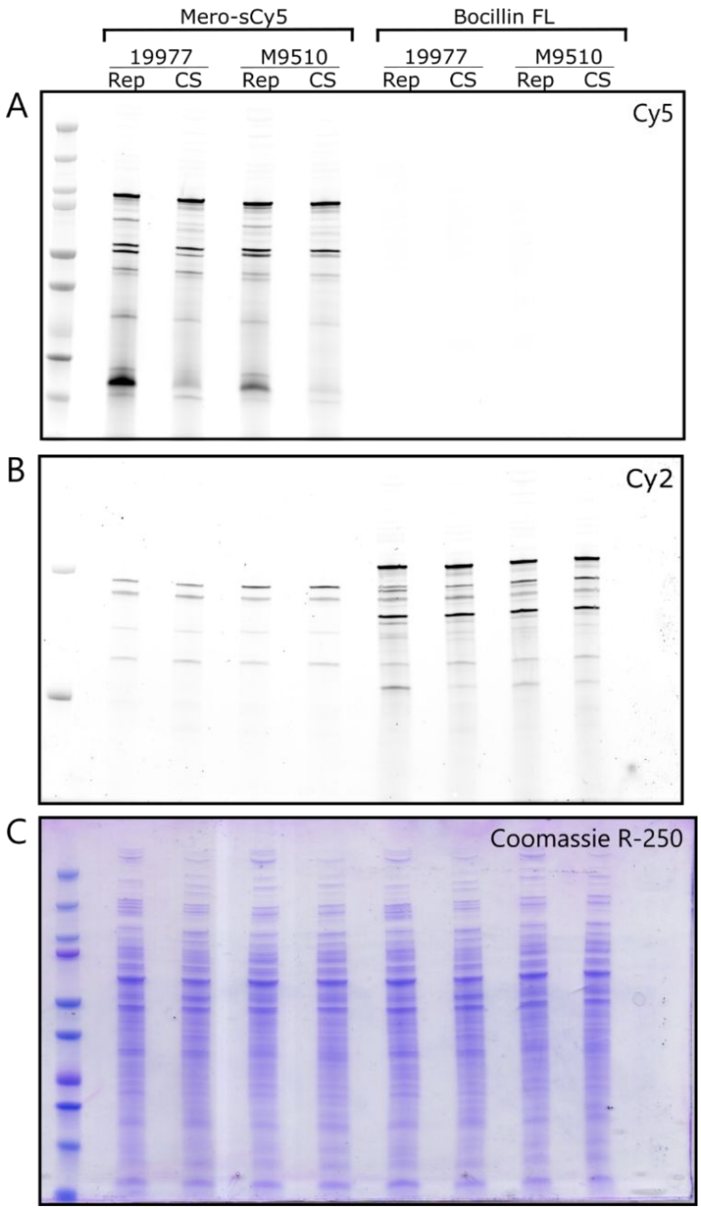

Figure S3. Β-lactam probe labeling of *Mab* lysates. Meropenem-sCy5- and Bocillin FL-labeled 19977 and M9510 lysates (12 µg) were resolved by SDS-PAGE and scanned for fluorescent signal in the Cy5 and Cy2 channels. Mero-sCy5-labeled samples showed signal in the Cy5 channel (**A**) with no visible autofluorescence. Bocillin FL-labeled samples fluoresced in the Cy2 channel (**B**) with several autofluorescent bands, as seen in samples not labeled with Bocillin FL. Equivalent loading across samples was verified through staining of total protein (**C**) by Coomassie R-250.

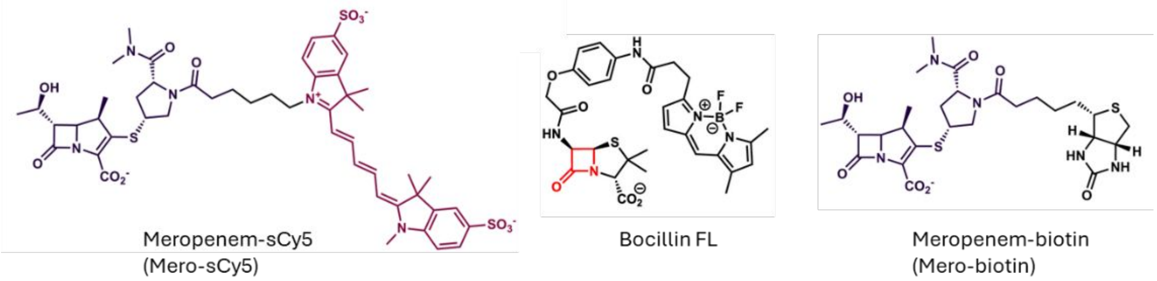

Figure S4. Structures of ABPs used in the current work.

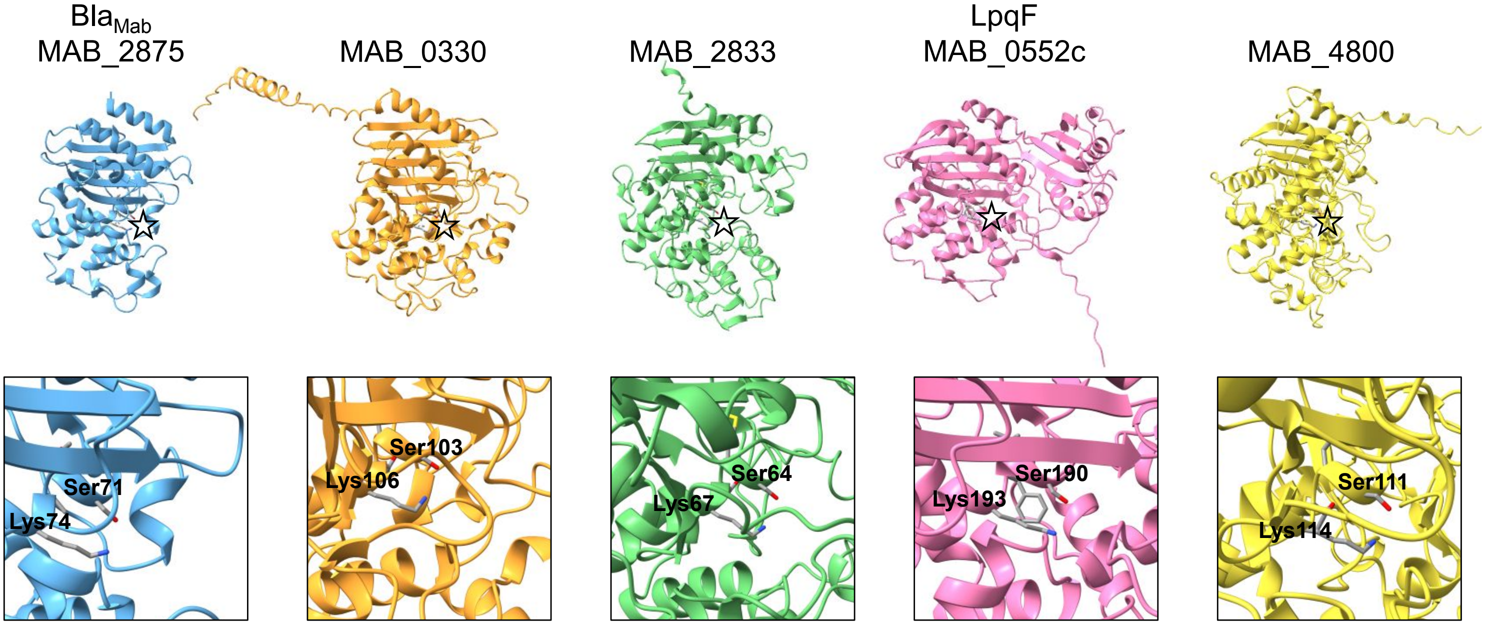

Figure S5. Structures of known and predicted β-lactamases in *Mab*. Top: Ribbon diagrams of the known (Bla$_{Mab}$; PDB ID 4YFM) and predicted (Alphafold[24]) protein structure of each enzyme. Black star indicates the active site residues. Bottom: Magnified view of the (predicted) active site (Ser-X-X-Lys). Structures were analyzed in ChimeraX[25].

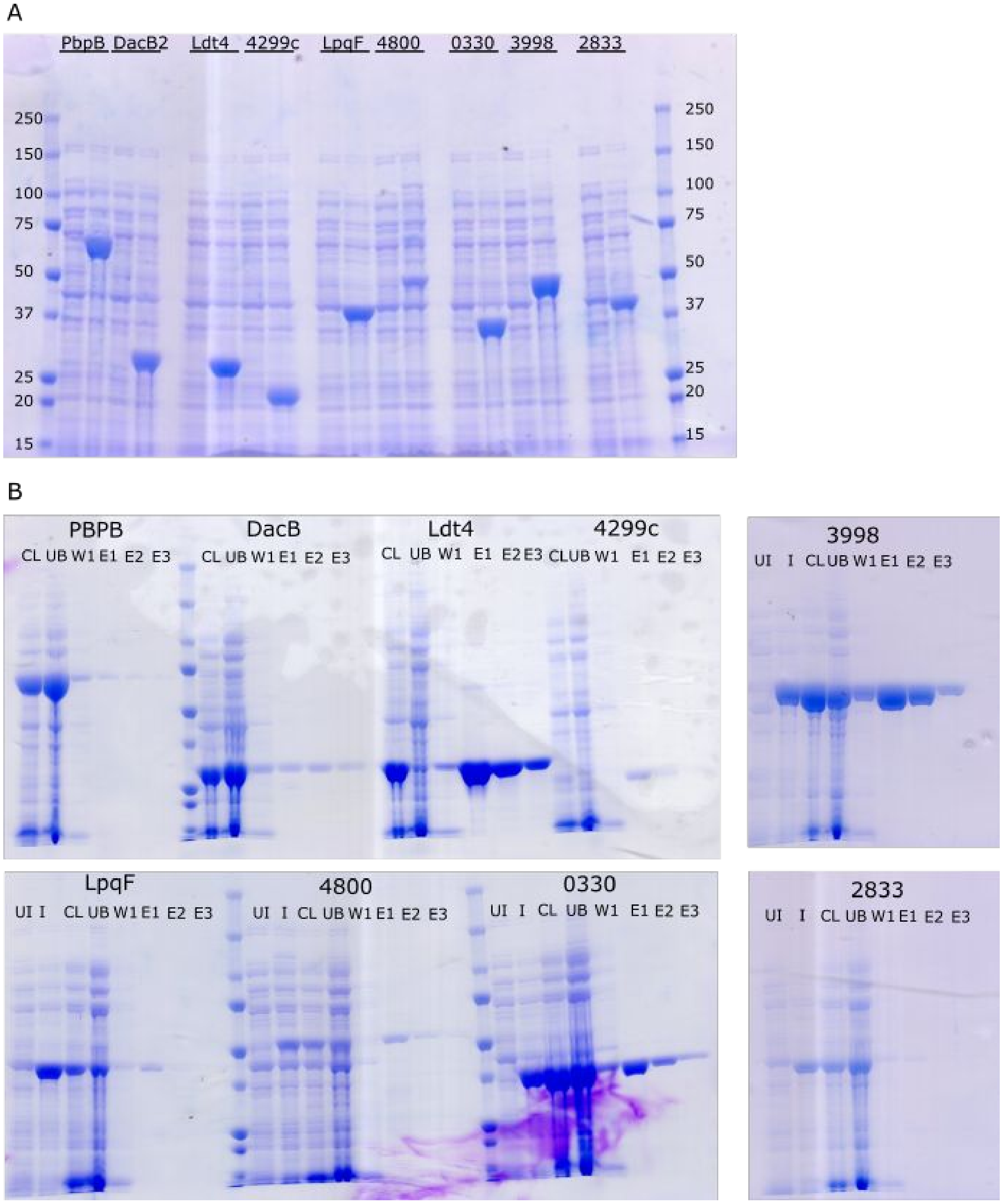

Figure S6. Gel analysis of *Mab* enzyme expression and purification. *Mab* enzymes were expressed in *E. coli* BL21-Star-DE3 cells and natively purified. **A**) Following expression, samples of uninduced (first lane) and induced (second lane) transformed cells were lysed in 6 M urea and analyzed by SDS-PAGE. Robust expression of each enzyme is observed at the appropriate molecular weight (Table S4). B) Fractions from enzyme purification were collected and analyzed by SDS-PAGE. Fractions analyzed were as follows: UI, uninduced; I, induced; CL, clarified lysate; UB, unbound by resin/flowthrough; W1, wash 1; E1-3, elutions 1-3.

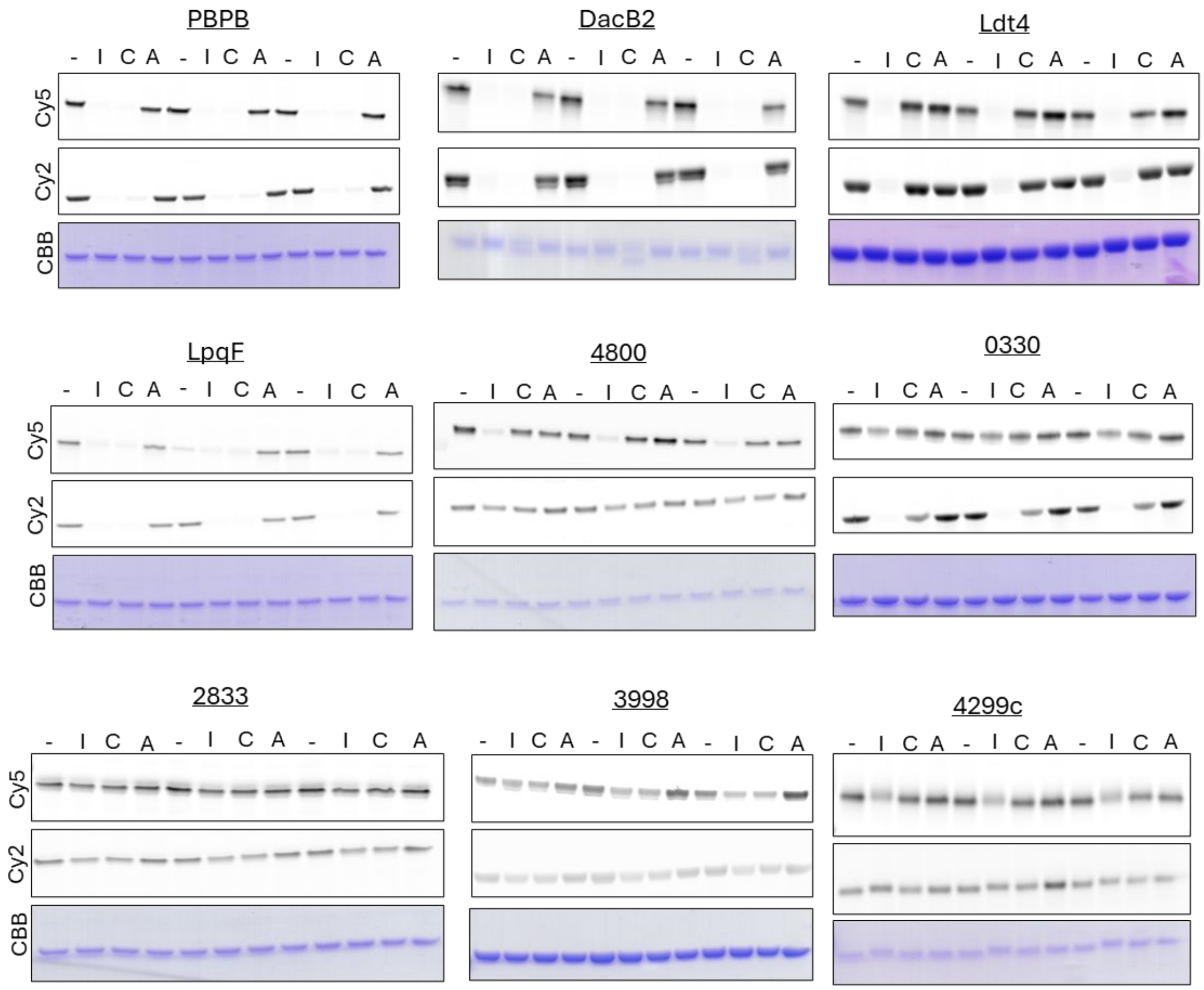

Figure S7. In-gel analysis of β-lactam probe labeling of *Mab* enzymes. Purified recombinant enzyme (3 µg) was combined with drug (500 µM; I: imipenem, C: cefoxitin, A: avibactam) or buffer (-) in 10 mM HEPES pH 7.5 (30 min, 37 °C). Samples were labeled with Mero-sCy5 or Bocillin FL (10 µM) (1 hr, 37 °C). Samples were prepared in triplicate and resolved via SDS-PAGE (1 µg/lane). Gels were scanned for fluorescent signal (Cy5: Mero-sCy5, Cy2: Bocillin FL) and stained with Coomassie R-250 (CBB) for total protein visualization.

A

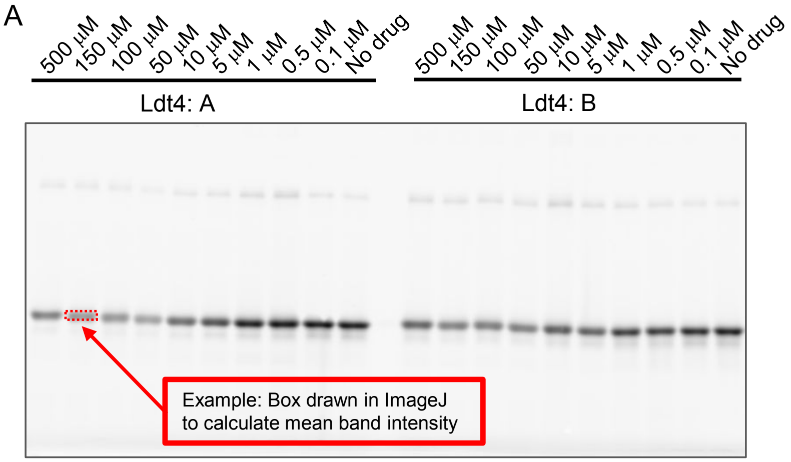

B

	Mean Band Intensity	Mean Band Intensity
[IPM]	LDT4_A	LDT4_B
500	10839.27	13318.44
150	9196.464	10944.07
100	8951.75	11278.36
50	8211.185	10886.55
10	12469.61	13755.81
5	14639.7	13101.44
1	18281.81	15630
0.5	18902.52	15657.46
0.1	18659.24	16905.22
0	18297.62	17978.04

C

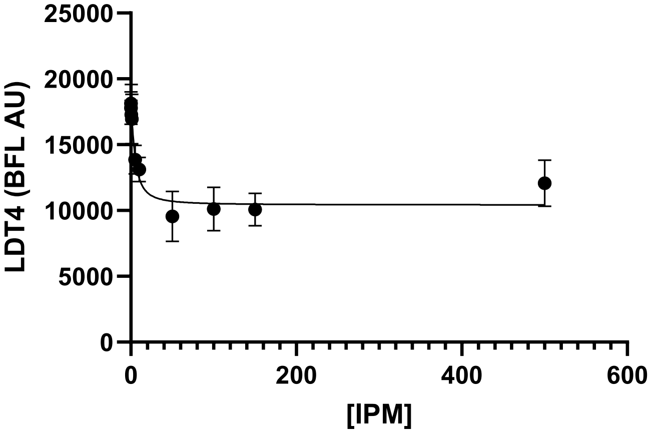

D

[Inhibitor] vs. response --
Variable slope (four parameters)

Best-fit values	
Bottom	10423
Top	17660
IC50	5.176
HillSlope	-1.416
logIC50	0.7140
Span	7236
95% CI (profile likelihood)	
Bottom	8895 to ???
Top	16528 to 19627
IC50	1.796 to 11.10
HillSlope	-5.428 to 0.5631
logIC50	0.2542 to 1.045
Goodness of Fit	
Degrees of Freedom	16
R squared	0.8570
Constraints	
IC50	IC50 > 0
Number of points	
# of X values	20
# Y values analyzed	20

Figure S8. Method used for IC50 determination for Ldt4. A. Fluorescent scan of SDS-PAGE gel including Ldt4 enzyme pre-treated with imipenem (500 - 0.1 µM) or no drug and labeled with Bocillin FL. **B.** Mean band intensity (in arbitrary units, au) was measured in Image J. **C.** A dose response curve was plotted in GraphPad Prism. **D.** Linear regression analysis was used to determine the IC50.

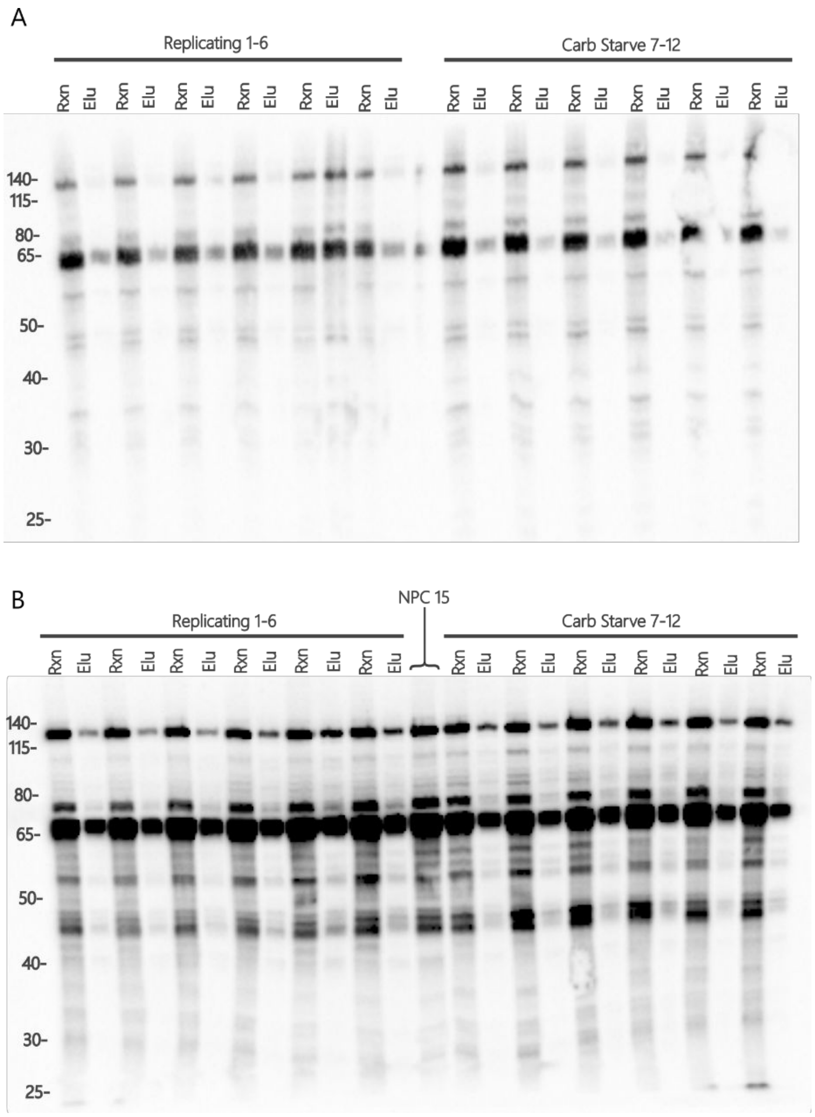

Figure S9. Western blot analysis of ABPP samples. Probed (Mero-biotin) and unprobed (NPC) total protein samples from *Mab* 19977 (**A**) and M9510 (**B**) were analyzed by anti-biotin western blot. Samples were normalized by protein amount (6 µg). Fractions from samples post labeling (Rxn) and after binding to streptavidin resin (Elu) were included.

Supplementary Tables

Table S1: *M. abscessus* culture media recipes.

Medium	Component	Supplier	Final Concentration
**LGM1**	Middlebrook 7H9 broth	BD Difco #271310	4.7 g per 1 L
	Glycerol (molecular biology grade)	Fisher	0.5%
	Tween 80	Sigma	0.05%
	BSA fraction V (molecular biology grade)	RPI	0.5%
	D-Dextrose, anhydrous	Sigma	11.1 mM
	Sodium chloride	Sigma	13.8 mM
	H_2O		
**LGM1-Tx**	Middlebrook 7H9 broth	BD Difco #271310	4.7 g per 1 L
	Glycerol (molecular biology grade)	Fisher	0.5%
	Tyloxapol	Sigma	0.05%
	BSA fraction V (molecular biology grade)	RPI	0.5%
	D-Dextrose, anhydrous	Sigma	11.1 mM
	Sodium chloride	Sigma	13.8 mM
	H_2O		
**7H9-Tx**	Middlebrook 7H9 broth	BD Difco #271310	4.7 g per 1 L
	Tyloxapol	Sigma	0.05%
	H_2O		
**7H11-S**	BBL 7H11 agar	BD #212203	9 g per 1 L
	Glycerol (molecular biology grade)	Fisher	0.25%
	BSA fraction V (molecular biology grade)	RPI	0.5%
	D-Dextrose, anhydrous	Sigma	11.1 mM
	Sodium chloride	Sigma	13.8 mM
	H_2O		

Table S2: *See corresponding Excel file "**ESI Table S2_Mab mero-biotin hits**".*

Table S3: *Mab* proteins with endogenous biotinylation or biotin binding, based on *M. tuberculosis* homology.

Name	*Mab* Locus ID	*Mtb* Locus ID	MW (kDa)	Protein Function
AccA1	MAB_4539c	Rv2501c	68.74	Biotin carboxyl carrier protein and biotin carboxyltransferase.
AccA2	MAB_1071c	Rv0973c	72.77	Biotin carboxyl carrier protein and biotin carboxyltransferase.
AccA3	MAB_3643	Rv3285	63.11	Biotin carboxyl carrier protein and biotin carboxyltransferase.
AccD1	MAB_4540c	Rv2502c	56.41	Component of the acetyl coenzyme A carboxylase complex.
AccD2	MAB_1072c	Rv0974c	56.50	Involved in fatty acid metabolism.
AccD4	MAB_0181	Rv3799c	56.38	Key enzyme in the catabolic pathway of odd-chain fatty acids, isoleucine, threonine, methionine, and valine.

AccD5	MAB_3631	Rv3280	59.03	Key enzyme in the catabolic pathway of odd-chain fatty acids, isoleucine, threonine, methionine, and valine.
AccD6	MAB_1876c	Rv2247	50.12	Involved in fatty acid biosynthesis (mycolic acids synthesis).
AccE5	MAB_3632	Rv3281	9.96	Involved in long-chain fatty acid synthesis.
BioA	MAB_2688c	Rv1568	47.21	Involved in bioconversion of pimelate into dethiobiotin. Supposedly involved in stationary-phase survival.
BioB	MAB_2684c	Rv1589	36.96	Involved in biotin synthesis.
BioD	MAB_2686c	Rv1570	23.10	Involved in bioconversion of pimelate into dethiobiotin.
BioF	MAB_2687c	Rv1569 / Rv0032	40.01	Involved in biotin biosynthesis.
BirA	MAB_3626c	Rv3279c	27.96	Biotin-operon repressor and enzyme that synthesizes the co-repressor, acetyl-CoA:carbon-dioxide ligase. Activates biotin to form biotinyl-5'-adenylate and transfers the biotin moiety to biotin-accepting proteins.
BisC	MAB_3041	Rv1442	84.34	This enzyme may serve as a scavenger, allowing the cell to utilize biotin sulfoxide as a biotin source.
PCA	MAB_3267c	Rv2967c	120.88	Involved in gluconeogenesis and lipogenesis. Catalyzes a 2-step reaction, involving the ATP-dependent carboxylation of the covalently attached biotin in the first step and the transfer of the carboxyl group to pyruvate in the second.
Tb7.3	MAB_3541c	Rv3221	7.40	Function unknown.

Table S4: *See corresponding Excel file "**ESI Table S4_19977 v M9510 hits**".*

Table S5: Summary of *Mab* protein constructs.

Enzyme	Locus ID	UniProt ID	Amino Acid Sequence‡<br>Key: His tag; TEV recognition; recombinant protein; proposed active site	MW (kDa)	Vector name
**Bla$_{Mab}$**	MAB_2875	B1MCI3	MGSSHHHHHHSSGENLYFQGHMAPDELASLEKDFGGRIGVYALDTGSGDTVGHR<br>ADERFLMCSTVKTFIVSAILRRRLSEPGLLDQRIQYTQSDVLEWAPITSQHVSTGMT<br>VSELCDATLRYSDNTGANLLITQLGGPKETEKFVRSLGDNVTRMDRTEVQLNIPDG<br>DLDTSTPQQLVANLRRLVLDEGLDSRGRDLLTDWLKRNTTGDQSIRAAVPAGWT<br>VADKTGGGFKGETNDIAVIWPPGRAPIVMAVLTVPEDPTSTKGKPTIAAATRIVLRA<br>FGA	30.56	pET28a_His6-TEV-BlaMab(31-289)
**DacB2**	MAB_3234	B1MDJ1	MGSSHHHHHHSSGENLYFQGHMDMAAPPPEGPAQAWLVADLDSGQILAARDPY<br>ATHAPASTIKVLLALVALDEVPMDATVVADAADAKAECNCVGIKAGQTYTARELL<br>DAALLESGNDAANTLAHLLGGRQATVDKMNAKAAALGATSTHTDSPSGLDAPG<br>MDMRTSPHDLAVIFRAALANPVFAEITAQPSAPFPGRGLHNQNELMYRYPGVIGG<br>KTGFTDIARKTYVVAAERDGKRLVVSMMYGLVHEGGPTYWDQAASLFDWGFVND<br>GSSSVGSL	29.20	pET28a_His6-TEV-DacB2(32-287)
**PBPB**	MAB_2000	B1MP31	MGSSHHHHHHSSGENLYFQGHMGPRAAGLRAEASGQLKVTETEKALRGTIVDRA<br>GNKLAFTIEARALTFQPKKIREQLNKAWEKSQEILKDPGKSDADKAAAAKIRSVEP<br>ERRLQDIANGVSAKLGNKPDAKSLLKKIRSDDTFAYLARSVDPGIAAQITKEFPEV<br>GAERQDIRQYPGGSLAANIVGSIDWEGYGLLGLEDSLDSALAGKDGSLTYDRGSD<br>GAVIPGSYRDRHDAVNGSTVELTLDNDIQFYVQQQVQQAKDLSEARSVSAVVLDS<br>KTGEVLAMANDNTFDPSQNLGKQGNREMGNLSVSSPFEPGSVNKVITASAVIEND<br>LSNPDEVLQVPGSINMGGVTVRDAWPHGTVPFTTTGVFGKSSNVGTLMLAQRVG<br>PERFMDLVDKFGLGQRTGVGLPGESAGIVPPIEQWSGSTFSNLPIGQGLSMTLLQ<br>MAGMYQAIANDGVRIPPRIVKSITAPDGSRKEEPRPEPVQVVNAGTARTVRNMLR<br>AVLQPDARQIQNGTGASGAVDGYQLSGKTGTAQQINPACGCYFDDVYWITFAGIA<br>TTDDPRYVIGIEMNAPKRGSDGSPHMTAAPLFHNIASWLMRRENVPLSPDPGPPLI<br>LQAT	65.27	pET28a_His6-TEV-DacB2(55-642)
**LDT4**	MAB_4537c	B1MKW1	MGSSHHHHHHSSGENLYFQGHMGDVPPLPVGLLKNSPNNGDVVGVAQPITIVFAA<br>PVTDKKAAEAAVKITTSKPAPGYFYWYTDQQLRWKPTQFWPANTDVNVNAGGTK<br>WSFKVGDAFVSTVDDATYTMTVTRNGVVERTMPISMGKHDKKHETKNGTYYVSE<br>KFQKMVMDSSTYGVPVNSAEGYKLDVYWATRLSNSGIFVHAAPWSVGAQGKSD<br>TSHGCINVNTDNATWFFNQSHPGDPVIVKNSPGGPYKDYDGYDDWQRF	29.15	pET28a_His6-TEV-Ldt4(92-333)
**LpqF**	MAB_0552c	B1MGW7	MGSSHHHHHHSSGENLYFQGHMSTPPGLRSQQLIDMLNSNWPIGKQGVATMAA<br>ANKVDDYTEIMTKLWVDRPYKVSSVDLGANSSTVHLIAPYGANVDIGLRTNDKGD<br>VDRLVPYQQPPTIANWSDVDAALSASGGRYSYRASKIVDGQCQQIAGTNTSQAM<br>PLASVFKLYVLLAAGTAINAGTLRWDDQLTVTDRGKALGSSMDKLPTGSTVSVRT<br>AAQKMISVSDNMGTDMLINRVGRHAIEKALADAGHHDPASMTPFPTMHELFNIGW	45.89	pET28a_His6-TEV-LpqF(47-446)

			GIPDVRQQWKDATTPEQRGRMLAEADTHDYKLDPDRTTTPASKYGIEWYGSAEDICRVHAALQKVAVGPAAPVRDILAAEPGIDLHSENWRYIGAKGGNLPGDLTFSWYAEDRTRQPYVVSFQLNWDRAFGPGAAGWVIGLAKGVFKKLG		
**MAB_0330**	MAB_0330	B1MFM9	MGSSHHHHHHAELDPQTTAKLDSAINEILTSAAVPGAIVGVWGPKGQYVRTFGVANTATRAPLRPDFYHRIGSVTKTFTVTAVLQLVDEGKVELDDPIAKYVDKVPNGNKITLRELARMQSGLFNYMSLAFSRDLEADPRRQYSTHELLDYAFAQPSNFVPGQGYEYSNTNAVLLGQVVEKVSGQTLPAFVQEHITGPLGMRRTSFPETSAIADPHARGYTQLTPTGSLVDSTDWNPSWASWAGAMISTLDDLRIWVPALATGVLLKPDSQQQRLQTVAMPPVPDDIRYGLGIFDARGWIGHSGSIPGYQTLAIYSPAEQTTVVALLNTDVAKQPAQPSTLFGTAITKVISPDHVFLLENAIPQPR	39.88	pET28a_His6-TEV-0330(41-397)
**MAB_2833**	MAB_2833	B1MCE1	MGSSHHHHHHSSGENLYFQGHMAEPDSTFDAALAPIRAAIDDRILAGAVTLVWQGGQLRHLGATGYRDIDAGLSMAENTIFRIASMTKPIISAATMNLVDDGTIRLSDPITTWLPEFSDMRVLKNPEGPLDDTFRAPRLITVEDLLTHRSGLTYDFISTGPIAKAYHPLHTAAFREPDEWLAAIAALPLVYPPGERFHYSHSTDVLGLLIARAAGLPLNTLLRQRILDPLGMNDTDFFVPEHKAARLARLYGLGDDDTIVAADSGYLTAMPTSAPALCRGGGALASTAHDYLTFARALLGGGQADGVRILSPESTQALRTNRLTPAQRRLPSFGIPYWTGRGFGLGLSVVMDPNEAALFGPGGTGTFGWPGAFGTWWHADPKADAILMFLPQWRMPELDPKAALARTSTIRLQLLHVQFGQAVYAAL	46.26	pET28a_His6-TEV-2833(1-406)
**MAB_3998**	MAB_3998	B1MHJ1	MGSSHHHHHHSSGENLYFQGHMSKEPGSQKLSADEAKSIAMDAYVYGYALVTMEMTRRVMTNVEKAEPPRAPMGQLMRMREYPNAAFRDVTAPNADTLYTNGFIDVGKEPWILSLPEADGRYYLFPMLDGFTNVFEVPGKRTTGTGPQTYAITGPGWKGTLPQGVQEYKSPTALVWLLGRIYCTGTPEDYAAVHTLQDAISLVPLSSYGKPYTPAAGHVDPSIDMTTPVREQVNNLSTKAYFDLLATLLKDNPPAEADKPILEKMATIGIEPGKPFDTDKLGTQTVSALESVPKEAFAKIMAHFKEAGNNINGWVFTTKTGQYGTDYLQRALITAIGLGANRPQDAVYPTSEVDNTGKPYDGANKYVLHFDKGQFPPAEGFWSLTMYDAGMFFVDNPLGRYTLSQRNTFTPNPDGSVDLYLQHQNPGPEKEANWLPAPTGKFNLMLRLYWPKETPPSIIDGTWKPPAVQQVP	52.15	pET28a_His6-TEV-3998(26-477)
**MAB_4299c**	MAB_4299c	B1MJL5	MGSSHHHHHHSSGENLYFQGHMEPFVPWFAQSVGNATQVIAVNGAGGSNAKIDVFQRNGSQWQTVSTGIPAHVGSAGFIDKAKEGASATPNGVYSLDWAFGTAPPPPSGLRYLQVGPNDWWDGDSNSPTYNTHQQCAKAECPFNTAQSENLPIPQYKHAIVMGVNKDRVPGGGSAFFVHSTDGGPTAGCVSLDDATLVKLIGWLRPGAVIAIKG	22.60	pET28a_His6-TEV-4299c(27-218)
**MAB_4800**	MAB_4800	B1MM83	MGSSHHHHHHSSGENLYFQGHMGRPAQPITAPSIVPTGPFAPITTLVNDAVAAPRLPGAVVEIGHAGKIVFRQAFGWRKLPDEPGLNGSPAPAEPMTDDTIFDIASLTKPLATSVAVLQLYEGGRVHIDEPVQAYLPDFNPTNDPRRDQVTLRMLLTHTSGIGGDLSHQGPWGLKQADKADGIHRALTTPLAFDPGTTFHYSDINFIILGALVEKVTGQTLDIYVQDNIFAPLGMNETRYLPAAKACGPHHIVGTAITLEEGPRAAPDCATGAWSTALLTRIAPTAHDEDTPGINPHYGRLIRGTVHDPTARRMGGVAGSAGVFSTAGDVGRYCQALLDRLAGRPSTFPLKRATLELMTSPAQPGHNDGQLKAANDAARLAIEKTSNSTDPLLAPGYPAIAGQNLRGLGWDIDTELSKPRGMLFPIGSFGHSGFTGVTLWLDPGSDTYVVVLANVIHQRGGPPIVRLSGHIATVAARMLDLYRN	51.05	pET28a_His6-TEV-4800(28-489)

References

(1) Nicklas, D. A.; Maggioncalda, E. C.; Story-Roller, E.; Eichelman, B.; Tabor, C.; Serio, A. W.; Keepers, T. R.; Chitra, S.; Lamichhane, G. Potency of Omadacycline against Mycobacteroides abscessus Clinical Isolates In Vitro and in a Mouse Model of Pulmonary Infection. *Antimicrob Agents Chemother* 2022, 66, e0170421.
(2) Story-Roller, E.; Galanis, C.; Lamichhane, G. beta-Lactam Combinations That Exhibit Synergy against Mycobacteroides abscessus Clinical Isolates. *Antimicrob Agents Chemother* 2021, 65.
(3) Berube, B. J.; Castro, L.; Russell, D.; Ovechkina, Y.; Parish, T. Novel Screen to Assess Bactericidal Activity of Compounds Against Non-replicating Mycobacterium abscessus. *Front Microbiol* 2018, 9, 2417.
(4) Lee, J.; Ammerman, N.; Agarwal, A.; Naji, M.; Li, S. Y.; Nuermberger, E. Differential In Vitro Activities of Individual Drugs and Bedaquiline-Rifabutin Combinations against Actively Multiplying and Nutrient-Starved Mycobacterium abscessus. *Antimicrob Agents Chemother* 2021, 65.
(5) Yam, Y. K.; Alvarez, N.; Go, M. L.; Dick, T. Extreme Drug Tolerance of Mycobacterium abscessus "Persisters". *Front Microbiol* 2020, 11, 359.
(6) Devlin, K. L.; Hutchinson, E.; Dearing, H. N.; Levine, S. R.; Reid, D. J.; Leach, D. T.; Griggs, L. H.; Lomas, G. X.; Gorham, L. J.; Wright, A. T.; et al. Comprehensive

Identification of beta-Lactam Antibiotic Polypharmacology in Mycobacterium tuberculosis. *ACS Infect Dis* 2025, 11, 2422-2433.
(7) Schindelin, J.; Arganda-Carreras, I.; Frise, E.; Kaynig, V.; Longair, M.; Pietzsch, T.; Preibisch, S.; Rueden, C.; Saalfeld, S.; Schmid, B.; et al. Fiji: an open-source platform for biological-image analysis. *Nat Methods* 2012, 9, 676-682.
(8) Monroe, M. E.; Shaw, J. L.; Daly, D. S.; Adkins, J. N.; Smith, R. D. MASIC: a software program for fast quantitation and flexible visualization of chromatographic profiles from detected LC-MS(/MS) features. *Comput Biol Chem* 2008, 32, 215-217.
(9) Kim, S.; Pevzner, P. A. MS-GF+ makes progress towards a universal database search tool for proteomics. *Nat Commun* 2014, 5, 5277.
(10) Stratton, K. G.; Webb-Robertson, B. M.; McCue, L. A.; Stanfill, B.; Claborne, D.; Godinez, I.; Johansen, T.; Thompson, A. M.; Burnum-Johnson, K. E.; Waters, K. M.; et al. pmartR: Quality Control and Statistics for Mass Spectrometry-Based Biological Data. *J Proteome Res* 2019, 18, 1418-1425.
(11) Jeong, K.; Kim, S.; Bandeira, N. False discovery rates in spectral identification. *BMC Bioinformatics* 2012, 13, S2.
(12) Astrand, M. Contrast normalization of oligonucleotide arrays. *Journal of computational biology : a journal of computational molecular cell biology* 2003, 10, 95-102.
(13) Gautier, L.; Cope, L.; Bolstad, B. M.; Irizarry, R. A. affy—analysis of Affymetrix GeneChip data at the probe level. *Bioinformatics* 2004, 20, 307-315.
(14) Polpitiya, A. D.; Qian, W.-J.; Jaitly, N.; Petyuk, V. A.; Adkins, J. N.; Camp, D. G., II; Anderson, G. A.; Smith, R. D. DAnTE: a statistical tool for quantitative analysis of -omics data. *Bioinformatics* 2008, 24, 1556-1558.
(15) Boekweg, H.; Payne, S. H. Challenges and Opportunities for Single-cell Computational Proteomics. *Molecular & Cellular Proteomics* 2023, 22, 100518.
(16) Webb-Robertson, B.-J. M.; McCue, L. A.; Waters, K. M.; Matzke, M. M.; Jacobs, J. M.; Metz, T. O.; Varnum, S. M.; Pounds, J. G. Combined Statistical Analyses of Peptide Intensities and Peptide Occurrences Improves Identification of Significant Peptides from MS-Based Proteomics Data. *Journal of Proteome Research* 2010, 9, 5748-5756.
(17) The UniProt, C. UniProt: the universal protein knowledgebase. *Nucleic Acids Res* 2017, 45, D158-D169.
(18) Emms, D. M.; Kelly, S. OrthoFinder: phylogenetic orthology inference for comparative genomics. *Genome Biol* 2019, 20, 238.
(19) Li, L.; Stoeckert, C. J., Jr.; Roos, D. S. OrthoMCL: identification of ortholog groups for eukaryotic genomes. *Genome Res* 2003, 13, 2178-2189.
(20) Steinegger, M.; Soding, J. MMseqs2 enables sensitive protein sequence searching for the analysis of massive data sets. *Nat Biotechnol* 2017, 35, 1026-1028.
(21) Camacho, C.; Coulouris, G.; Avagyan, V.; Ma, N.; Papadopoulos, J.; Bealer, K.; Madden, T. L. BLAST+: architecture and applications. *BMC Bioinformatics* 2009, 10, 421.
(22) Alexeyenko, A.; Tamas, I.; Liu, G.; Sonnhammer, E. L. Automatic clustering of orthologs and inparalogs shared by multiple proteomes. *Bioinformatics* 2006, 22, e9-15.
(23) Kocaoglu, O.; Carlson, E. E. Profiling of beta-lactam selectivity for penicillin-binding proteins in Escherichia coli strain DC2. *Antimicrob Agents Chemother* 2015, 59, 2785-2790.

(24) Jumper, J.; Evans, R.; Pritzel, A.; Green, T.; Figurnov, M.; Ronneberger, O.; Tunyasuvunakool, K.; Bates, R.; Zidek, A.; Potapenko, A.; et al. Highly accurate protein structure prediction with AlphaFold. *Nature* 2021, 596, 583-589.
(25) Meng, E. C.; Goddard, T. D.; Pettersen, E. F.; Couch, G. S.; Pearson, Z. J.; Morris, J. H.; Ferrin, T. E. UCSF ChimeraX: Tools for structure building and analysis. *Protein Sci* 2023, 32, e4792.
